# Raman spectroscopy identified fingernail compositional differences between sexes and age-related changes but not handedness or fingers in a healthy cohort

**DOI:** 10.1371/journal.pone.0329092

**Published:** 2025-08-22

**Authors:** Nai-Hao Yin, Frances Griffiths, Claire Mann, Helen Dawes, Richard van Arkel, Marwan Bukhari, Jemma G. Kerns

**Affiliations:** 1 Lancaster Medical School, Faculty of Health and Medicine, Lancaster University, Lancaster, United Kingdom; 2 Warwick Medical School, The University of Warwick, Warwick, United Kingdom; 3 College of Medicine and Health, University of Exeter, Exeter, United Kingdom; 4 Department of Mechanical Engineering, Imperial College London, London, United Kingdom; 5 Department of Rheumatology, University Hospitals of Morecambe Bay NHS Trust, Lancaster, United Kingdom; Harvard School of Public Health, UNITED STATES OF AMERICA

## Abstract

**Background:**

Nail properties and appearances can indicate a person’s underlying systemic diseases. Raman spectroscopy is an established laboratory technique and has been applied to nails, identifying spectral differences between healthy individuals and patient populations.

**Objective:**

We aim to explore the importance of potential spectral or chemical variations in nails between sexes, age groups, hands, and fingers.

**Methods:**

Twenty male and twenty female participants without known musculoskeletal or dermatological diseases donated nail clippings from each finger. The clippings were cleaned, and Raman spectra collected and analysed using a standardised protocol.

**Results:**

In total 2000 spectra were collected. Females have higher intensities of disulphide, protein, and lipid bands, particularly in their 40s, than males. Age-related changes were prominent in female nails, especially in sulphur-related bands. No significant differences were observed between nails from the left and right hands or among different fingers.

**Limitations:**

We did not control other factors such as diet, medication, or different occupation or sports participation.

**Conclusion:**

This is the first study to use Raman spectroscopy to compare nail composition across different ages and sexes in healthy adults. The findings provide a strong basis for further studies on nails at the population level for screening or monitoring diseases.

## Introduction

While nail diseases are common but usually affect appearance more than function, the change in nail properties or appearances can sometimes indicate systemic diseases originating from systems other than dermatological [1,2]. For example, shape changes like koilonychia or clubbing can indicate iron-deficiency anaemia [3,4] and cardiopulmonary diseases [5], and colour changes could suggest circulatory problems [6,7] or heavy metal poisoning [8]. More recently, nails have been used as an easily accessible tissue in some cross-sectional studies and the biochemical composition has been associated with breast [9], stomach [10], and colon [11] cancers. Nail appearances, pigmentation, and the quantity of trace elements showed differences among paediatric [12] and adult [9,13] patients undergoing chemotherapy. Studies also suggested that fingernail compositions differ between osteoporotic and non-osteoporotic patients [14] and between diabetic and non-diabetic patients [15]. While nails appearances are part of medical assessments, utilising compositional analysis of nails has not been fully explored but could form a new approach for diagnosis and/or screening of systemic diseases.

There are few studies comparing fundamental differences between nail compositions of both sexes in different ages or between different hands or fingers. It has been suggested that among healthy individuals, the nitrogen, carbon, sulphur, magnesium, and calcium content [16–18] as well as the keratin structure and composition of fingernails [19] could be different between sexes and can change with age. In a study conducted on individuals living in non-industrial environments, the concentration of trace elements showed different patterns with age while males had higher sodium and potassium concentrations than females [20]. Surprisingly, there are only two studies that reported the potential nail differences between left and right hands and between different fingers. It has been reported that inter-individual variability is higher than intra-individual differences for water content [21] and transonychial water loss [22] of nails from different fingers. To the best of our knowledge, no study has compared biochemical or compositional differences between fingernails.

Raman spectroscopy is an established laser-based laboratory technique that allows tissue composition at the molecular level to be probed with minimal sample preparation [23,24]. Combined with the easily accessible nature of fingernail clippings, Raman spectroscopy has the potential for rapid, large-scale screening of nail compositional differences at the population level. Several studies have been conducted using this approach to differentiate spectral features between asymptomatic individuals and patients [14,25,26]. However, most of the studies have utilised either arbitrarily selected or any random fingernail, without the acknowledgement of any (or lack of) potential compositional differences between fingers or hands. Without a robust study, it is not valid to assume that different fingernails have the same composition since the mechanical stimuli are likely different between the left and right hands and, for example, between the thumb and little fingers. For some studies, with a focus on female participants only, it is also not known whether there are sex differences between males and females due to hormonal influences and/or aforementioned mechanical differences. This fundamental knowledge needs to be addressed before we can fully utilise spectroscopy techniques to monitor diseases with nails.

This study aims to identify whether Raman spectroscopy can identify spectral, and therefore chemical, differences between and within the nails of healthy males and females, and whether age-related variations are present.

## Materials and methods

### Participants

This study was approved by Lancaster University Faculty of Health and Medicine Research Ethics Committee (FHM-2024–4089-RECR-2) and advertised through internal communications and posters. The recruitment period started on the 1^st^ of March and ended on the 30^th^ of June 2024. The inclusion criteria were adults over 18 years old and without any diagnosed major systemic, dermatological diseases or musculoskeletal injuries to the hand, wrist, or forearm. After received written consent, at least one fingernail clipping from each finger was collected from each participant.

In total 40 participants aged between 22 and 63 participated. The sex and age distribution are reported in Table 1, with four age groups selected for further sub-group analysis.

**Table 1:** Age and sex distribution of participants.

	20–29 yrs	30–39 yrs	40–49 yrs	50–63 yrs
Male	4	8	5	3
Female	4	8	5	3
Total	8	14	10	6

### Sample collection and preparation procedure

The clipped nails were stored in sealed plastic bags from collection until spectroscopic measurement. The nail clippings were cleaned with an acetone-based solution (Superdrug Beauty Essentials Nail Polish Remover, Product code: 219148, Superdrug Stores plc) and agitated for 30-sec and left to dry for at least 30 min at room temperature before spectral acquisition. All the nail clippings were aligned transversely to the laser with the anterior distal side facing upwards. The testing sequence for each participant was based on the recruitment process and the order was randomised for different fingers.

### Raman spectroscopy

A Renishaw inVia Raman spectrometer (Renishaw, Gloucestershire, UK) equipped with an Olympus 50 × /0.5 long working distance objective lens and 785 nm laser was used for all the measurements, with 5-sec exposure time and 6 accumulations under 100% laser power (200 mW at source) in the spectral range of 450 cm^-1^ to 1800 cm^-1^. Spectra were collected from five randomly selected spots, at least 10 μm apart, in the middle of the sample to account for the heterogeneity of biological tissues. Fifty spectra (5 spectra by 10 fingers) were collected for each individual. A total of 2000 spectra were collected.

### Data analysis

All the analysis was performed in MATLAB (R2023b, The Mathworks, Inc., Natick, MA, USA) with a previously published script for spectral analysis [27,28]. The raw spectra were baseline corrected using a 5^th^ order polynomial and normalised to the phenylalanine peak (1002 cm^-1^). The following group comparisons were made using multivariate statistical analyses: males vs. females, different age groups (20–29, 30–39, 40–49, > 50 yrs), left vs. right hands, and among 5 fingers in each hand. Principal component analysis (PCA) was performed to objectively evaluate the group differences. In addition, PCA with linear discriminant analysis (LDA) was performed using known labels to evaluate whether spectral differences exist between different groups. Principal component scores, loadings and group means were used to visualise spectral differences.

## Results

### Differences between male and female nails

**Fig 1:**
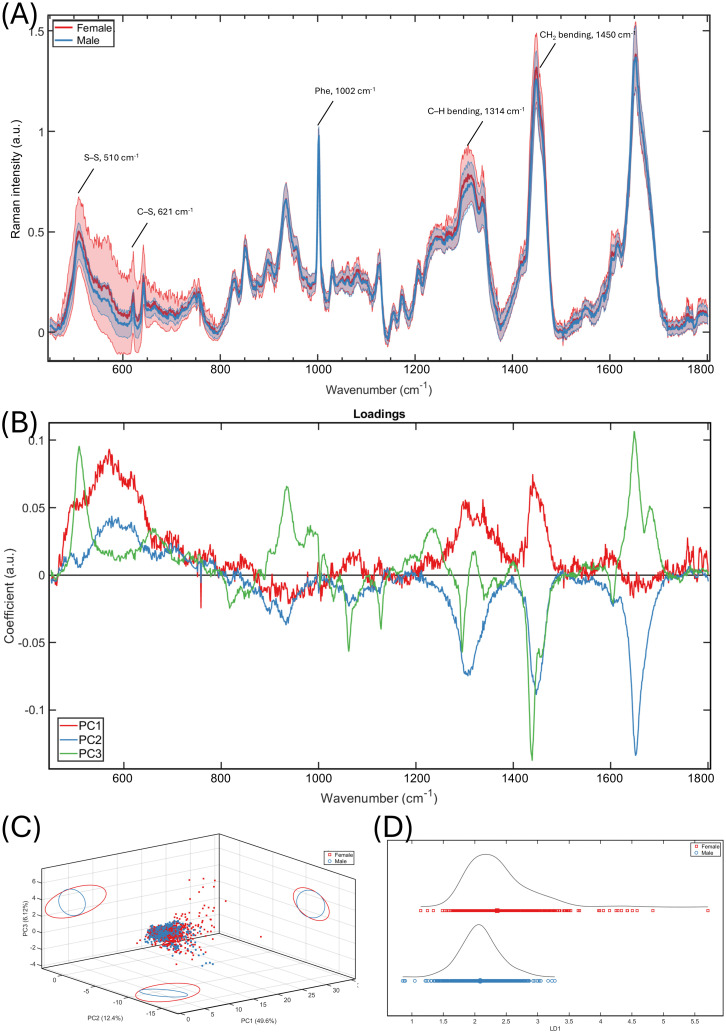
Raman spectral differences between both sexes. **(A)** Mean spectra and standard deviation of female (red, n = 20) and male (blue, n = 20) participants. **(B)** The loading graph of the first three principal components. **(C)** 3D scatter plot (female: red, male: blue) with projection of 95% confidence interval of the first three principal components. **(D)** Linear discriminant analysis of the first 10 principal component of both sexes.

In general, we were able to collect high-quality data from nails with clear keratin Raman spectral features (Fig 1A). Differences in Raman spectra between both sexes were present in the mean spectra and the multivariate analyses. The mean Raman spectra of female fingernails showed higher intensities and larger standard deviation than males in the wavenumber region between 450 cm^-1^ and 800 cm^-1^, primarily related to sulphur bonds, and between 1300 cm^-1^ and 1350 cm^-1^, attributable to protein structure or lipids (Fig 1A). Observing the mean spectra (Fig 1A) and the PC1 loadings from principal component analysis (Fig 1B) suggested that the band S–S stretching (510 cm^-1^) and C–S stretching (621 cm^-1^) modes, C–H bending mode (1314 cm^-1^), and CH_2_ bending (1450 cm^-1^) band had higher intensities in females than males. PCA scores plot suggested that PC1 and PC2 had larger confidence interval in female spectra than male (Fig 1C) and LDA separated the sexes, as observed in the scores plot (Fig 1D).

**Fig 2:**
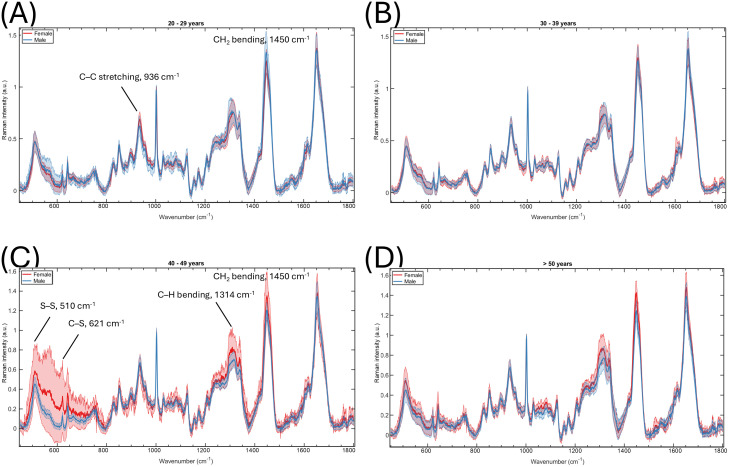
Mean and standard deviation of Raman spectra of both sexes grouped by different ages. **(A)** 20 to 29 years old, n = 8. **(B)** 30 to 39 years old, n = 14. **(C)** 40 to 49 years old, n = 10. **(D)** over 50 years old (51–63 yrs), n = 6. Female indicated by red lines, male by blue.

When grouped by different ages, the spectral differences between sexes are less prominent in the younger age groups than in the older groups (Fig 2). The females in their 20s (Fig 2A, red) had higher C–C stretching (936 cm^-1^) but lower CH_2_ bending (1450 cm^-1^) bands than males while there was no visible difference in the spectral means of both sexes in the 30s group (Fig 2B). The most prominent sex differences in the mean Raman spectra were found in the 40s group (Fig 2C) with the females having higher intensities in the sulphur-related wavenumber region (< 800 cm^-1^), wavenumbers between 1250 cm^-1^ and 1350 cm^-1^, and CH_2_ bending band at 1450 cm^-1^ than males. The sulphur region also showed a large standard deviation which suggests high individual differences in this particular age group, especially among females. The spectral differences between sexes were less prominent in the oldest age group while the general trend remained similar to the 40s (Fig 2D).

### Differences between different age groups

**Fig 3:**
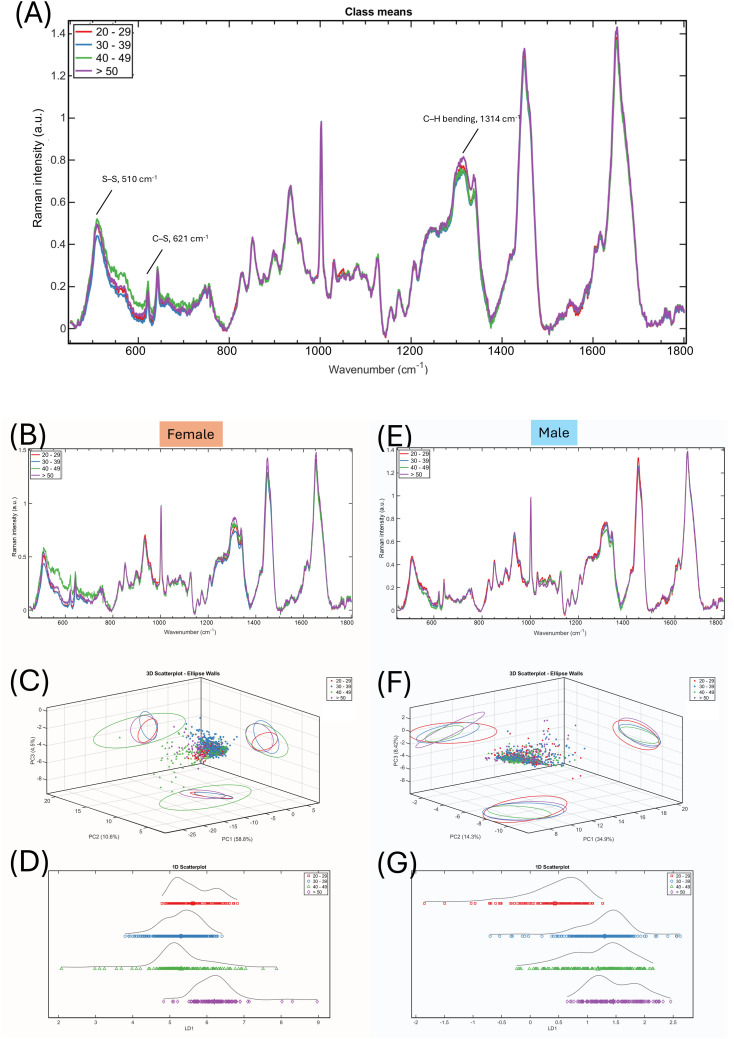
Mean and standard deviation of Raman spectra of four different age groups. **(A)** Spectra from all participants (n = 40). **(B – D)** Spectra mean of female participants (n = 20), the 3D scatter plot of principle component analysis, and the linear discriminant analysis. From top to bottom: 20–29 yrs, red; 30–39 yrs, blue; 40–49 yrs, green; and > 50 yrs, purple. **(E – G)** Spectra mean of male participants (n = 20) and the multivariate analyses results.

When pooled for both sexes, age-related spectral differences are mostly observed in the sulphur region with the S–S stretching (510 cm^-1^) and C–S stretching (621 cm^-1^) bands in addition to the C–H bending mode (1314 cm^-1^, Fig 3A). When separating female (Fig 3B–3D) and male spectra (Fig 3E–3G), the age-related changes were more prominent in female fingernails compared to males, especially in the 40s group. The aged-related change for male was not evident from subjectively observing the spectral means or objectively from the multivariate statistical analyses (Fig 3E–3G).

### Differences between left and right hands and between fingers

**Fig 4:**
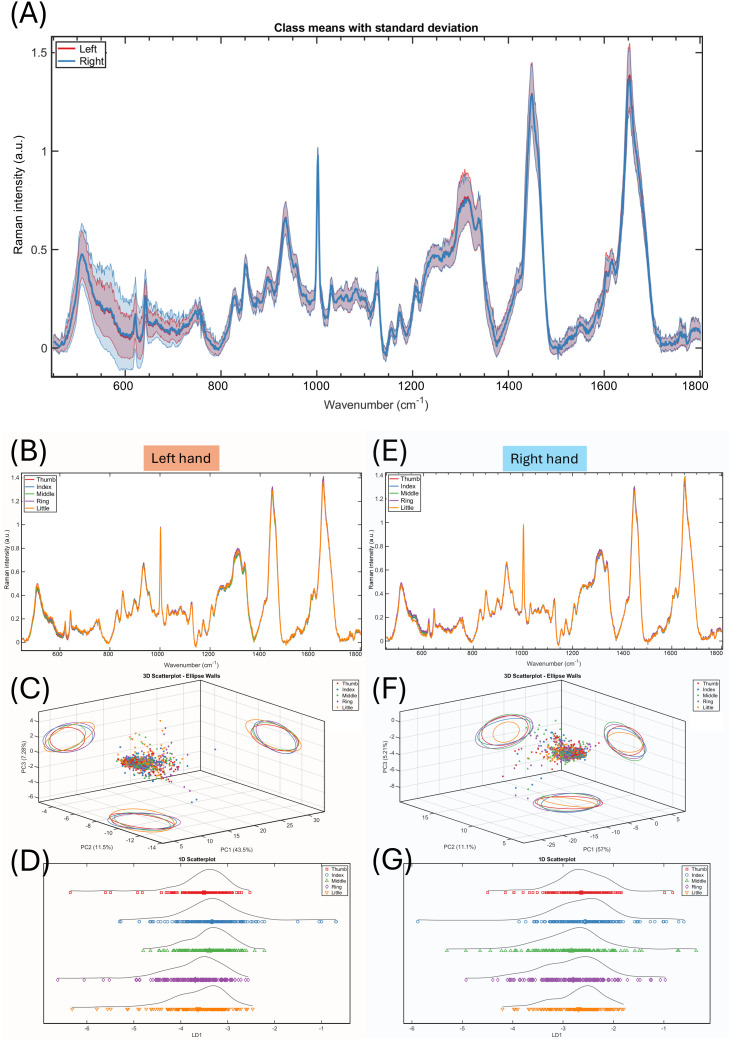
Raman spectra grouped by hands and fingers. **(A)** Mean spectra and standard deviation of left (red) and right (blue) hands. **(B – D)** Spectra mean from fingers of the left hands, the 3D scatter plot of principal component analysis, and the linear discriminant analysis. From top to bottom: thumb, red; index, blue; middle, green; ring, purple; and little, orange. **(E – G)** Spectra mean from fingers of the right hands and the multivariate analyses results.

There were no discernible differences between the Raman spectra collected from the left or the right hands (Fig 4A). Similarly, there were no significant or consistent differences among fingers on either left (Fig 4B–4D) or right hands (Fig 4E–4G).

## Discussion

We have demonstrated that nail compositions, measured by Raman spectroscopy, are different between female and male participants and show age-related differences. These differences are predominantly found in chemical bonds related to sulphur (500–800 cm^-1^) and carbon-hydrogen bonds (centred at 1314 cm^-1^ and 1450 cm^-1^). Interestingly, when analysing spectra grouped by both sex and age, it appears that the greatest sex differences are found in the 40s group, and females generally showed more prominent age-related differences compared to males. On the other hand, we found no differences between left and right hands or among fingers, suggesting that handedness or habitual use may have little influence on nail compositions.

Our collected Raman spectra of fingernails are in accordance with previous reports, which utilised nail clippings from unspecified finger(s) [18,25,29]. Widjaja et al [18] collected 240 Raman spectra in total from 40 participants and they suggested that observed sex differences are attributed to amino acids cysteine, phenylalanine and tyrosine. Brzózka et al. [19] reported that, by using nuclear magnetic resonance spectroscopy, the keratin secondary structure and the amino acid composition are different among healthy young males and females. The authors concluded that the high variability between individuals is contributed from the disulphate bond in the cysteine residues and can be used for discriminating different sexes, in accordance with our Raman spectroscopy findings in the sulphur-related wavenumber region. Using biochemical approaches, Dittmar et al. [16] reported that female nails have more sulphur and nitrogen than males while ageing was associated with a reduction in nitrogen but an increase in carbon content. These organic component changes are likely to influence the keratin composition. With age, calcium concentration in nails decreases but magnesium increases for both sexes, and these concentrations were correlated with lumbar bone mineral density of the sub-group of participants in their 60s [17]. It is currently not known whether hormonal, diet or other environmental influences can also cause the observed differences between females and males.

Similarly, we found age-related differences in our Raman spectra and the differences were more prevalent in female participants than males, especially in the 40s age group. It is possible that a reduction in oestrogen associated with the menopause can affect the nail protein structures since the effect was not observed in our male participants [30,31]. Nail changes (fragile, brittle, or peeling) are commonly reported symptoms in menopause and linked to oestrogen deficiency [30,31], and it is reported that nail capillary blood flow is decreased in post-menopausal women but can be improved by hormone replacement therapy [32]. Studies conducted on post-menopausal women suggested the quantity of disulphate bonds of keratin (bands centred at 510 and 621 cm^-1^) in nails could be different between osteoporotic and non-osteoporotic participants [14,25,26]. Animal studies have also demonstrated significant changes to the nail and claw chemical compositions during puberty [33] and following ovariectomy [34]. It is known that hormonal influence is systemic and can impact on musculoskeletal tissues [35]. Previous studies using spectroscopic approaches demonstrated that changes in protein structure can precede to significant tissue changes, such as in bones [36,37] or tendons [38].

Ageing can also result in increased protein glycation. Veras et al [15] collected nail spectra from 60 diabetic and non-diabetic participants aged between 45 and 65 years old and found that diabetic patients had a higher lipid content but lower disulphate bonds, with a change in protein secondary structure. The authors suggested that these changes are detrimental to the mechanical strength of the nails in diabetic patients. Our results in male participants showed an age-related decline in the 1450 cm^-1^ band, which is often associated with lipid content or total protein levels and requires further investigation. With a limited number of participants over 40 years old (n = 16), we cannot draw a firm conclusion from our results; however, this warrants investigation and future studies should focus on exploring the age and sex differences in nail compositional changes, with particular focus on menopause status.

Previous reports have indicated that dominant and non-dominant hands can have small (~5%) but significant differences in phalangeal bone mineral density using dual-energy X-ray absorptiometry [39]. Their results also suggested that the difference is consistent with sex and age, and is likely a direct result of mechanical stimuli. We did not record the dominant side of our participants but found no significant differences in the spectral features between left and right hands. In addition, we found no observable spectral features between different fingers, which are likely subjected to different mechanical stimuli throughout the day (such as thumb and index versus ring and little fingers), but how these mechanical stimuli translate into significant compositional, therefore spectral, difference in nails remains to be studied. To the best of our knowledge, signs of systemic disease are unlikely to appear on one particular fingernail or predominantly left or right hands, regardless of handedness. However, our current approach with Raman spectroscopy did not study the mechanical properties or the water content in nails that may have differences between dominant and non-dominant hands.

Based on our findings, future studies using nail clippings as a surrogate tissue for monitoring or screening systemic diseases should be able to use the clipping of any single finger from either hand, as we found no significant difference in the spectra, hence the molecular level composition of nails, among different hands and fingers. With an appropriate sample size and large spectral dataset across different ages and both sexes, we demonstrate the potential of analysing nails and provide a basis for further studies on nails at the population level for screening or monitoring diseases.

There are a few limitations that prevent further interpretation of the results. Firstly, we did not control for other factors that could potentially change the nail compositions either by systemic influences (such as diet or medication) or local mechanical stimuli (such as occupation or sports). Whether these differences contribute to any observable change in the collected Raman spectra among individuals is unknown and should be explored further to fulfil the potential of this technology for screening or monitoring diseases. It is also important to note that distal nail clippings are exposed to environmental influences, and therefore may have some compositional differences, compared to the proximal fingernail bed. Any potential chemical or mechanical differences, and the resulting Raman spectral changes, between the clipped and the attached fingernails remains to be explored. Secondly, our participants were more skewed towards the younger age groups and while we excluded those with diagnosed musculoskeletal and dermatological diseases, there is a chance that some participants are asymptomatic and could therefore influence the collected spectra. Lastly, there could be other differences between sub-groups that our current spectroscopic approach cannot detect, such as the water content in the nails or the fluorescence intensities, which was removed at the first step during the standardised data treatment process.

## Conclusions

Our study demonstrated that nails are a suitable tissue for Raman spectroscopy assessment of individual differences, and confirms, for the first time, that future studies can use any nail to be representative of the individual since minimal intra-individual compositional differences exist between hands and fingers. Care should be taken when comparing Raman spectra between sexes and between different ages, as there could be fundamental spectral and compositional differences that need further exploration. Future study incorporating a larger sample size and controlling potential confounding factors to provide baseline information on Raman spectral features of nail clippings from different sexes and age is warranted.
