# Supplementary material for: Raman spectroscopy identified fingernail compositional differences between sexes and age-related changes but not handedness or fingers in a healthy cohort
Source: PLoS One. 2025 Aug 22;20(8):e0329092. doi: 10.1371/journal.pone.0329092 (PMC12373161; doi:10.1371/journal.pone.0329092)
Supplement: Attachment — Submitted filename: Yin2025_ResponsetoReviewerComments.docx [file pone.0329092.s002.docx]

**We would like to thank the reviewers’ time and effort in reviewing our article.**

Reviewer #1: A major issue is whether the clipped nail truley reflects the status of the proximal attached nail. The end of nail is free to environment on both surfaces and possibly altered. In the situation where changes most marked a sample of nail (how many?) attached should be tested and compared to clipped nail.

**We appreciate the comment and agree that the distal end of fingernails is exposed and could subject to more environmental wear and tear compared to the proximal, attached nail. All nails were cleaned prior to analysis. We have added a sentence in the discussion to address the limitations of proximal nail analysis (Lines 279 – 282).**

Reviewer #2: The paper PONE-D-25-13367 explores the application of Raman spectroscopy on fingernails, aiming to detect compositional differences related to sex, age, handedness, and finger origin. The study is interesting and generally well conducted, however there are some issues to solve before publication, in particular to better clarify the novelty in relation to previous studies, actually the authors claim in the conclusions that “our study demonstrated that nails are a suitable tissue for Raman spectroscopy assessment”, suggesting novelty. However, they also state earlier that “our collected Raman spectra of fingernails are in accordance with previous reports”. Please, indicate better the novelty with respect the previous studies.

**We thank the reviewer for their comment and have modified the conclusion to better reflect the novelty of this study. Specifically, that there are high inter-individual differences but no intra-individual differences using Raman spectroscopy on all 10 clipped fingernails (lines 291 – 293).**

2) In figure Figure 1a the Raman spectrum of fingerprints is reported, however there are no assignment to the different Raman peaks. This point is fundamental to better understand the variation reported.

**Thank you. We have added the peak assignments accordingly.**

3) . Figures 2 and 3 indicate age-related differences, especially in females. However, the discussion lacks depth in explaining the biological basis for these trends. The observation that females in their 40s show the largest differences is intriguing and possibly related to perimenopausal hormonal shifts. This deserves a more structured interpretation supported by citations. However the number of samples is relatively low (5) to enter into the assignment and could generate some confusion to the reader and to the technique. Please explore this part and improve the discussion of the section.

**We have expended the discussion regarding the biological basis for the trends, particularly in relation to oestrogen and nail changes, and have added supporting evidence (Lines 230 – 237). We have added an additional sentence (Lines 247 – 250) to emphasise the limited number of samples from this age group and to suggest future research focusing on this particular demographic (pre-menopausal women).**
